# Hyaluronan Functions in Wound Repair That Are Captured to Fuel Breast Cancer Progression

**DOI:** 10.3390/biom11111551

**Published:** 2021-10-20

**Authors:** Cornelia Tolg, Britney Jodi-Ann Messam, James Benjamin McCarthy, Andrew Cook Nelson, Eva Ann Turley

**Affiliations:** 1 London Regional Cancer Program, Lawson Health Research Institute, London, ON N6A 5W9, Canada; Cornelia.Toelg@lhsc.on.ca; 2 Department Biochemistry, Schulich School of Medicine and Dentistry, Western University, London, ON N6A 5C1, Canada; bmessam@uwo.ca; 3 Department of Laboratory Medicine and Pathology, University of Minnesota, Minneapolis, MN 55455, USA; mccar001@umn.edu; 4 Masonic Cancer Center, University of Minnesota, Minneapolis, MN 55455, USA; nels2055@umn.edu; 5 London Regional Cancer Program, Lawson Health Research Institute, Department Oncology, Biochemistry and Surgery, Schulich School of Medicine and Dentistry, Western University, London, ON N6A 5C1, Canada

**Keywords:** hyaluronan, RHAMM, CD44, wound repair, breast cancer

## Abstract

Signaling from an actively remodeling extracellular matrix (ECM) has emerged as a critical factor in regulating both the repair of tissue injuries and the progression of diseases such as metastatic cancer. Hyaluronan (HA) is a major component of the ECM that normally functions in tissue injury to sequentially promote then suppress inflammation and fibrosis, a duality in which is featured, and regulated in, wound repair. These essential response-to-injury functions of HA in the microenvironment are hijacked by tumor cells for invasion and avoidance of immune detection. In this review, we first discuss the numerous size-dependent functions of HA and emphasize the multifunctional nature of two of its receptors (CD44 and RHAMM) in regulating the signaling duality of HA in excisional wound healing. This is followed by a discussion of how HA metabolism is de-regulated in malignant progression and how targeting HA might be used to better manage breast cancer progression.

## 1. Background

Decades ago, Dvorak concluded that cancer is spawned in chronic non-resolving wounds, implicating a role for the status of the host microenvironment in cancer initiation, and focusing attention on identifying the processes of wound repair that are coopted by mutant cells to initiate tumors and support their progression to metastasis [1]. Subsequent studies have identified the processes of inflammation and fibrosis, which are critical to efficient wound repair, as key microenvironmental factors that promote cancer progression [2,3,4]. Hyaluronan (HA) has emerged as an important extracellular matrix (ECM) regulator of inflammation and fibrosis in the setting of excisional wound repair [5,6,7,8,9,10,11], and this polymer is also implicated in breast cancer progression [5,8,12,13,14,15]. Here, we review the well–regulated inflammatory and fibrogenic functions of HA polymers and two HA receptors—CD44 and RHAMM (HMMR)—in cutaneous wound repair and consider evidence that these functions also contribute to the progression of breast cancer.

## 2. The Hyaluronome

The collection of genes that controls the synthesis, metabolism, and signaling properties of the tissue polysaccharide, HA, are collectively called the hyaluronome, and include HA synthases responsible for the production of HA, HA receptors, which bind HA and activate cellular signaling cascades; and hyaluronidases, which break the native HA polymer into fragments that differ from the native polymer in their signaling functions [5].

### 2.1. Hyaluronan

**Figure 1:**
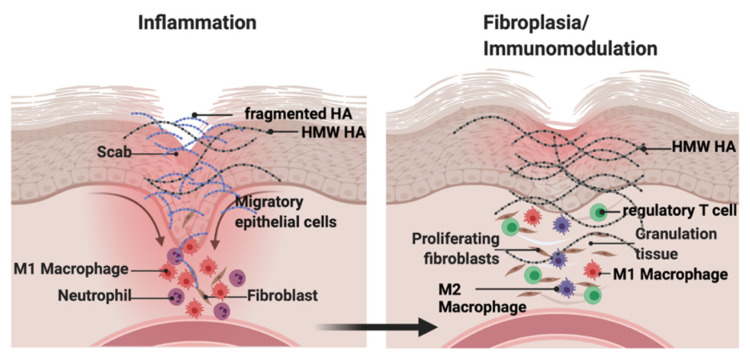
Hyaluronan contributes to inflammation and resolution of excisional wounds. Native (high molecular weight, HMW–HA) hyaluronan production is increased upon and throughout tissue injury. Low molecular weight (LMW–HA) and oligosaccharide (O–HA) fragments are rapidly generated from HMW–HA hyaluronidases and ROS/NOS, resulting in a mixture of HMW and fragmented HA polymers. LMW– and O–HA promote macrophage and fibroblast influx into the wound that initiate inflammation while HMW–HA restrains the extent of inflammation. At later stages of wound repair, HMW–HA predominates and supports macrophage polarization into the immunosuppressive M2 phenotype. Created with BioRender.com (accessed on 12 September 2021).

HA is a simple linear polysaccharide consisting of repeated saccharides (N–acetylglucosamine and B–glucuronic acid disaccharide units form the HA polymer), which was historically considered to be an ‘inert’ structural component. At that time, effects on cell behaviour and tissue homeostasis were postulated to result from the physicochemical characteristics of HA that provide tissue hydration, expansion and elasticity [16,17,18]. Although these physicochemical characteristics of HA are impressive and critical to the homeostasis of organs such as skin [19,20], the demonstration that HA activates kinase cascades in cultured fibroblasts [21] and binds to specific cell receptors such as CD44 [22] and RHAMM (HMMR) [23] provided initial evidence of its signaling properties. HA has since been shown to regulate MAP kinase, PI3 kinase, Hippo, and multiple growth factor signaling networks [5,24]. The complex functional information provided by this simple linear polymer is due in large part to metabolism related changes in both its expression level and in its molecular weight. For example, the native newly synthesized HA polymer (defined here as high molecular weight HA, HMW–HA, >500–700 kDa) blunts cell proliferation and migration and is anti–inflammatory as shown by studies demonstrating its functions to suppress an M1 and enhance M2 polarization of macrophages [14,25]. These properties of HMW–HA are considered important for maintaining tissue architecture and homeostasis particularly in skin. In contrast, smaller HA polymers created by enzymatic and/or chemical degradation of HMW–HA (e.g., low molecular weight HA, LMW–HA, 10–250 kDa; and oligosaccharides, O–HA, <10 kDa) function as ‘danger alerts’ (DAMPs [26,27]) that are released by cell death/stress and are strongly immunogenic. These tissue damage–induced HA oligomers provide pro–inflammatory (e.g., support M1 macrophage polarization), proliferation and migration signals [5,28,29] (Figure 1), and are critical for initiating a response to injury. It is intriguing that DAMPs released in response to tumor cell death, are also implicated in breast cancer progression [30], providing another example of the similarities between wounds and breast tumors.

### 2.2. Hyaluronan Synthases

In mammals, HA is synthesized by one or more of three genetically distinct cell membrane isoenzymes, hyaluronan synthases 1–3 (HAS1–3). These synthases differ in their enzymatic properties, regulation by stimuli, and contribution to normal and pathological processes. For example, whereas HAS1 and 2 synthesize HMW–HA (avg. 2 × 10^3^ kDa), HAS3 synthesizes shorter HA polymers (avg. 2 × 10^2^ kDa). HAS2 is expressed during early embryonic development and its genomic deletion leads to embryonic lethality resulting from cardiac defects [31]. In contrast, *Has*1^–/–^, *Has3*^–/–^, and *Has1*:*Has*3^–/–^ mice are viable [32,33,34]. HAS2 is expressed in most tissues including skin and mammary gland ductal epithelial and stromal cells [35], and elevated HAS2 expression has been linked to promoting breast cancer progression [13,36]. HAS1 and 3 are also expressed in the epidermis and dermis of the skin [37] although keratinocytes primarily express HAS3 [13,37,38] while dermal fibroblasts primarily express HAS1 [39,40]. All three HA synthases are upregulated during cutaneous wound repair and in some cancers [34,39] but, primarily, HAS2 is upregulated in breast cancer [41,42].

### 2.3. Hyaluronidases

HMW–HA produced by HAS1–3 is degraded into heterogeneously sized fragments by both hyaluronidases (HYALs) and cell metabolism by–products such as reactive oxygen and nitrogen species (ROS/NOS). HYALs are hyalurono–glucosidases that cleave the beta–(1,4) linkage between N–acetylglucosamine and glucuronate [43]. The human genome encodes *HYAL1–HYAL5* and one pseudogene (*HYAL6*). Out of these, HYAL1 (present in lysosomes) is mainly responsible for HA degradation into oligo–, di–, and monosaccharides while HYAL2 (localized to the cell surface via a GPI linker) degrades HA into fragments of about 20 kDa. These HYAL2 created fragments are either released into the microenvironment or internalized by HA receptors such as CD44 to be further degraded by HYAL1 [44,45,46]. Two additional proteins with hyaluronidase activity have more recently been discovered that process HA into intermediate–sized LMW fragments: the transmembrane protein TMEM2 [47] and KIAA1199 (CEMIP, HYBID) [48]. TMEM2 is a widely expressed membrane protein that can digest native HA into 5kDa fragments [47]. Pro–inflammatory cytokines that are released early in cutaneous repair such as IL–6, regulate KIAA1199, which is expressed by activated skin fibroblasts [49] and macrophages [50,51,52]. IL–6 and the resulting HA fragments [28,53] promote leukocyte infiltration into the wound. However, IL–6 also plays a key modulatory role in the switch from a pro–inflammatory to immunosuppressive microenvironment required for wound resolution [54]. The increased expression of IL–6 is also linked to breast cancer progression [48], where it performs both immunomodulatory functions similar to those in wound repair [55]. This cytokine has also been shown to modulate tumor cell plasticity, which impacts breast cancer progression and chemo–resistance [55]. Therefore, deeper analyses of the links between CEMIP and IL–6 are potentially important for understanding the commonalities between wound repair and cancer metastasis, as well as providing potential therapeutic targets to control both of these processes.

### 2.4. Hyaluronan Receptors, CD44 and RHAMM

To date, characterized HA receptors include CD44 [56], RHAMM (HMMR) [23], LYVE1 [57], TLR2/4 [29,58], STAB2/HARE [59], and LAYN [49]. CD44, LYVE1, and STAB2/HARE bind to HA via link modules [56,60]. In contrast, the HA binding domain of RHAMM has been localized to alpha helical clusters of positively charged residues [61,62]. TLR2 and 4 contain similar clusters of positively charged amino acids as RHAMM. Although these receptors are required for responses to HA fragments there is controversy as to whether or not they directly bind to this polysaccharide [60]. LAYN has been shown to bind directly to HA but contains neither link module nor clusters of positively charged amino acids, and the sequences responsible for this interaction have presently not been reported [5,28]. In this review, we focus upon the biology of CD44 and RHAMM because—unlike LYVE1, STAB2/HARE, and LAYN—these receptors have been studied in detail in the context of cutaneous wound repair and breast cancer [8] and because they are well characterized to directly bind to HA [60]. Readers interested in the biology of these HA receptors are directed to additional reviews [15,59,63,64,65,66,67].

Although CD44 and RHAMM clearly differ in key biochemical and structural properties, some commonalities include a likely evolution from heparin–binding ancestors, complex functions due to isoform expression generated by alternative mRNA splicing and coordinated intracellular and extracellular functions. CD44 is a non–kinase cell surface HA receptor that contributes to the proliferation, migration/invasion, adhesion, polarity, plasticity, and differentiation of many cell types, including resident skin cells [68,69]. CD44 is constitutively and widely expressed in tissues such as skin, and binds to HMW, LMW, and O–HA via a link module, which is distinct from the HA binding sequences of RHAMM [61,70]. The signaling properties of CD44 result not only from its association with HA polymers but also its interactions with other cell surface and extracellular proteins (e.g., growth factor receptors, osteopontin, metalloproteinases, and collagens) [71]. The small, intracellular domain (ICD) of CD44 additionally binds to intracellular adaptor and cytoskeletal proteins [72]. The binding of HA polymers to CD44 promotes homotypic CD44 clustering that can activate or impede oncogenic signaling cascades depending upon the HA polymer size and its partnering with other proteins. As an example, HMW–HA stimulates tumor–suppressive Hippo signaling by clustering CD44, which recruits polarity–regulating kinase (PAR1b) to the intracellular domain of CD44 and leads to activation of Hippo signaling [73]. In contrast, LMW–HA inhibits this Hippo signaling by disrupting HMW–HA/CD44 clustering. However, the interaction of CD44 with HMW–HA [74] and RHAMM can also result in expression of genes such as MMP9 that are utilized for both cutaneous wound repair and breast cancer progression [71].

**Figure 2:**
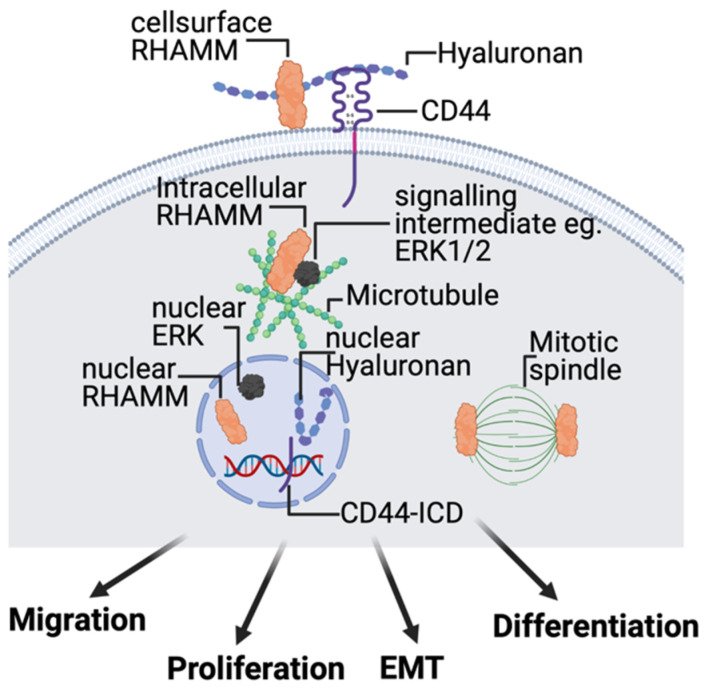
HA receptors CD44 and RHAMM regulate signaling that control skin cell migration, proliferation, plasticity, and differentiation during response–to–injury. CD44 is an integral membrane protein that coordinates signaling through growth factor receptors (e.g., EGFR) and cell surface RHAMM. The intracellular domain (ICD) of CD44, which can be released under injury conditions, forms part of transcriptional complexes that regulate expression of injury response genes. RHAMM also occurs in multiple intracellular compartments including the microtubule and actin cytoskeleton and, like CD44, is a component of transcriptional complexes regulating expression of extracellular matrix proteins that are required for wound repair. Created with BioRender.com (accessed on 12 September 2021).

In contrast to CD44, RHAMM expression is low and primarily intracellular in most homeostatic tissues but expression and extracellular export increases with pathologic stress, injury, and neoplastic transformation [8]. Under injury conditions, small amounts of intracellular RHAMM are released from cells, which bind to LMW and O–HA via alpha–helical clusters of positively charged amino acids [8,61,75]. The three–dimensional organization of these clusters is similar to those located in the alpha–helical glycosaminoglycan binding sites of lectins (e.g., GRO cytokines) [76,77]. RHAMM: HA complexes associate with integral HA receptors such as CD44 and TLR4 to activate signaling cascades (Figure 2), initiating an early response–to–injury through the NRLP3 inflammasome and other signaling cascades [14,28,29]. The functional and physical association of RHAMM with CD44 is influenced by the presentation of HA polymers in both soluble and ECM–immobilized form [78]. These interactions regulate cell motility and gene expression. The intracellular functions of RHAMM are complex and multifunctional, and include regulation of microtubule stability, mitotic spindle dynamics, intracellular signaling complexes, and gene transcription (Figure 2). Collective study of CD44 and RHAMM signaling predict that binding preferences for HA polymer sizes (which regulate receptor clustering), as well as mode of HA presentation, are two mechanisms for how cells detect and differentially signal in response to HMW–, LMW–, and O–HA polymers.

## 3. Functions of Hyaluronan: Size Matters in Cutaneous Repair and Breast Cancer

**Figure 3:**
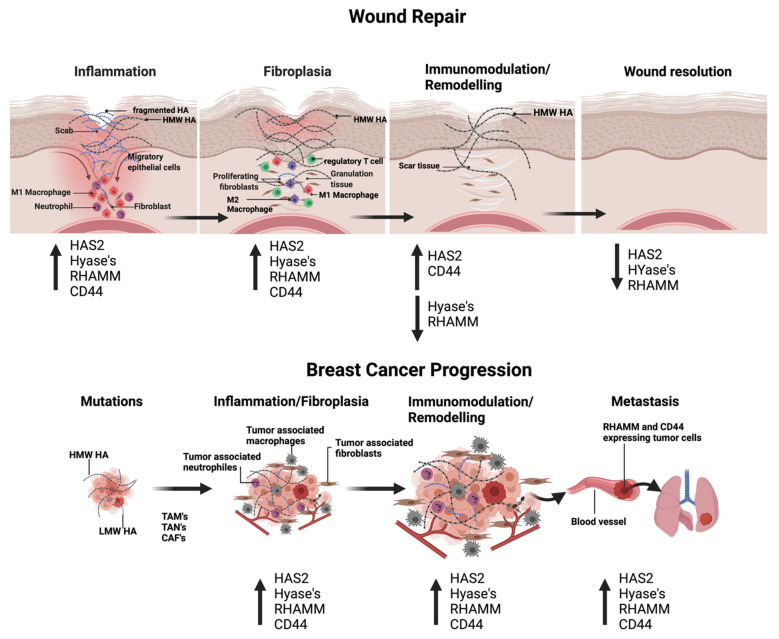
HA polymers, CD44 and RHAMM, regulate wound repair and promote breast cancer progression. During early stages of wound repair and during breast tumor progression, expression of HAS2, Hyal’s, CD44 and RHAMM is upregulated, providing optimal conditions for infiltration of immune cells and cell proliferation. During later stages of wound repair, Hyal and RHAMM expression is reduced, resulting in a prevalence of HMW HA and signaling via CD44. At the final stage of wound repair, HA production and HA receptor expression return to levels seen in unwounded skin. In contrast, HA synthesis, fragmentation and HA receptor expression remain high throughout breast cancer progression. Created with BioRender.com (accessed on 12 September 2021).

Are the functions of the hyaluronome in excisional wound repair replicated in breast cancer progression? While skin wound healing is a tightly regulated and orderly physiological response to injury, breast cancer is not. For example, cutaneous repair can be reproducibly simplified into three sequential stages: inflammation, fibroplasia, and the final immunomodulation/tissue remodeling required for wound resolution [79] (Figure 3). In contrast, during breast cancer initiation and progression, inflammation and fibroplasia are simultaneous and chronic with an evolution towards immunomodulation/remodeling that culminates in progression to metastasis (Figure 3). HA affects all three of the wound repair stages, particularly targeting immune and fibroblast functions (Figure 1). In general, HMW–HA is anti–inflammatory, anti–fibrotic, and pro–regenerative while HA fragments are pro–inflammatory, support fibroplasia, which results in scar formation in excisional wound repair [8,80] and alter the immune landscape of cancer microenvironments [15]. Nevertheless, some sizes of LMW–HA can be useful therapeutically since they can promote the rapid closure of wounds and reduce infection [15].

### 3.1. HA and Cutaneous Injury

In injured tissues, HMW–HA synthesis is closely coupled to the generation of LMW–HA fragments that initiate a robust inflammatory and fibrogenic response resulting in the rapid wound closure and control of opportunistic pathogens [81]. However, the reliance on inflammation as the initial response–to–injury results in a dermal scar that compromises skin elasticity and strength [25,80,82]. In contrast to adult tissue repair, embryonic wound repair, which occurs in a sterile environment, and proceeds in the absence of extensive HMW–HA fragmentation or immune cell influx, is regenerative, healing without a scar. Elevated expression of HAS1,2 by skin cells is responsible for the increased production of HMW–HA, which occurs throughout the repair stages [34,83]. ROS/NOS, in combination with released hyaluronidases, rapidly fragment a portion of the newly synthesized HMW–HA into a highly heterogeneous pool of LMW and O–HA polymers [81]. Thus, a mixture of HMW–, LMW–, and O–HA collectively contributes to the repair and resolution of excisional wounds.

The properties of HMW–HA perform multiple functions during excisional wound repair. HMW–HA provides a source for the generation of LMW– and O–HA polymer sizes, activates specific immunogenic signaling pathways and regulates fibrogenesis. As a source for generating HA fragments, HMW–HA contributes to inflammation. However, as a native polymer, it restrains HA fragment–induced inflammation by inhibiting MAP kinase, NFkB and other pathways, which blunt the expression of pro–inflammatory cytokines—such as TNFA, IL1B, IL–6, and CCL2 [84]—thereby suppressing the M1 pro–inflammatory polarization of macrophages [84,85]. HMW–HA also inhibits wound fibroblast expression of pro–inflammatory cytokines, e.g., IL–6 and other chemokines [86]. HMW–HA further contributes to dampening inflammation by promoting the polarization of M1 macrophages into an immunosuppressive M2 macrophage with the concomitant expression of cytokines such as TGFB1, IL10, IL11, and ARG1 [84]. These immunosuppressive cytokines are required for wound resolution [80,84,85,87]. For example, the HMW–HA/IL–10 axis affects adaptive immune response by modulating CD4+ effector T cells and promoting T regulatory cell function to reduce both innate immune activity and wound scarring [25,80]. In addition, HMW–HA reduces innate immune cell and fibroblast migration [88,89] and proliferation [90,91], which collectively control the extent of wound fibroplasia/fibrosis [92,93]. In contrast to these effects on immune cells and fibroblasts, HMW–HA (2290 kDa) stimulates keratinocyte migration and wound re–epithelialization [94]. These collective properties of HMW–HA have been utilized clinically to reduce inflammation and fibrosis tipping wound repair to a more fetal–like regenerative repair. Thus, injection of HMW–HA into keloids inhibits fibroblast proliferation [92], and reduces the fibrogenic properties of keloid fibroblasts [95] while topical application of HMW–HA [88] or forced overexpression of HAS1 to excisional skin wounds speeds repair [96] and reduces scarring [83].

In opposition to HMW–HA, LMW and O–HA fragments drive inflammation and fibroplasia during the early stages of excisional repair to enhance the speed of wound closure. Indeed, topical application of HYAL2 to full–thickness wounds speeds their closure [97]. SDS–PAGE analyses of wound and tumor HA reveal a continuous gradient of polymer sizes that is a complex mixture of biological cues to responding cells [5,81]. Studies have shown that HA fragments can have a precise size–dependent effect on excisional wound repair. For example, 40 kDa LMW–HA inhibits while 6mer O–HA (≈1 kDa) [98] and 250 kDa LMW–HA [99] promote wound closure in vivo, and selectively regulate expression of pro–inflammatory and immunosuppressive cytokines [100] as well as production of chemokines that attract fibroblasts into excisional wounds [101]. Although some HA polymer sizes have distinct functions during repair, others exhibit functional duality. For example, 500 kDa HA exerts both pro– and anti–inflammatory effects on macrophages [84]. This mixture of distinct and overlapping functions likely provides an exquisitely subtle control of inflammation and fibrosis. It is noteworthy that acute application of HYAL2 to full thickness skin wounds speeds wound closure [97]; continuous application of large amounts of O–HA to excisional wounds prevents wound repair [96], indicating that tight control of fragmentation is necessary for normal wound repair.

### 3.2. HA and Breast Cancer

Currently, there are no clear genetic abnormalities associated with the critical transition from DCIS to invasive cancer; however, there is emerging evidence linking this progression to tumor–induced changes in the microenvironment [3]. In particular, evidence supports a role for the immune/inflammation [102,103,104] and fibrogenic functions [12,105,106] of a wound–like host microenvironment in providing conditions to support early breast cancer cell spread and progression to a metastatic state. It is important to note that both host and tumor cells contribute to a cancer microenvironment, and that the evolution of a tumor–supporting microenvironment is chaotic in comparison to the defined stages of wound repair. Furthermore, tumor cells are highly plastic making both their contributions and responses to the microenvironment dynamic. These properties and the heterogeneity of breast cancers complicate efforts to dissect the roles of ECM components in breast cancer progression. Nevertheless, a change in HA metabolism has repeatedly emerged as one of the microenvironmental factors linked to breast cancer progression (Figure 3). For example, recent meta–analysis of published data from breast cancer patient tumors shows that increased HA accumulation in the tumor stroma [107] and LMW–HA in tumor patient plasma [108] are biomarkers for poor outcome. Experimental evidence shows that HA synthesis contributes to tumor supporting microenvironment [109], blocking HA production by knockdown of *HAS2* [110] and the use of inhibitors such as 4–Methylumbelliferone [111] inhibits tumorigenesis and metastasis of breast cancer cell lines. The concept that HA fragments fuel breast cancer progression is also supported by evidence that elevated *HYAL* expression (in particular CEMIP and TMEM2) is linked to breast tumor initiation [13]. However, the contributions of HMW–HA and LMW–HA to the wound–like inflammatory and fibrogenic properties of the breast cancer microenvironment support rather than resolve disease progression.

Like wounds, tumors contain a heterogeneous mixture of HMW–, LMW–, and O–HA polymers, which affect the function of both tumor and host cells [5]. However, unlike the coordinated synthesis and transient degradation of HMW–HA evident during wound repair [81], HA synthesis and HA fragmentation in tumors are deregulated, uncoupled and remain elevated during tumor progression with consequences to both tumor and host cells that support tumor progression rather than its resolution. For example, the beneficial functions of HMW–HA that facilitate wound resolution are co–opted by breast tumor cells to suppress immune detection and reduce exposure to therapy. Similar to its functions in wounds, HMW–HA promotes an immunosuppressive M2 macrophage polarization, particularly in the context of breast cancer [112,113]. While this function is essential for wound resolution, it contributes to signaling that supports immune evasion and progression of breast tumors [114]. For example, HMW–HA stimulates in–trafficking and primes tumor–associated macrophages to produce pro–angiogenic cytokines, which stimulates neoangiogenesis that contributes to disease progression [33,112]. HA also targets cancer–associated fibroblasts to promote their migration towards tumor spheroids, where their close proximity supports a paracrine tumor cell growth and migration [15,115,116]. The viscous properties of HMW–HA, which concentrate essential growth and other signaling factors near migrating cells to facilitate wound closure, also impedes therapeutic responses in cancer by reducing drug perfusion of tumors [117,118]. Two potentially tumor–suppressive effects of HMW–HA are its ability to arrest tumor cell proliferation [119,120] and increase breast tumor cell apoptosis [120]. However, the anti–proliferation function of HMW–HA is a two–edge sword since limiting tumor cell proliferation may actually attenuate the efficacy of cytotoxic chemotherapy that best targets proliferating cells.

Tumor cells, such as wound cells, detect and differentially respond to various sizes of HA fragments. The continual de–regulated synthesis of HMW–HA provides a constant source of LMW–HA and O–HA, which sustains host inflammation and fibrosis [13,14,121], and directly promotes breast tumor cell invasion and successful colonization of distant tissues [5,122], an event that does not happen during wound repair. LMW– and O–HA also promote expression of pro–inflammatory cytokines such as CCL2, which attract pro–tumorigenic circulating monocytes and stromal cells into the tumor microenvironment [33,123,124], and ECM regulators that support pro–tumor immunogenic and fibrogenic functions [125]. LMW–HA notably promotes invasion and migration of breast tumor cells, which is particularly observed in triple negative breast cancer [126,127]. Furthermore, triple negative breast cancer cell subpopulations that bind high levels of LMW–HA are more invasive and metastatic than tumor cells that bind only low levels [128]. Consistent with these experimental results, high levels of LMW–HA in the serum of breast cancer patients correlates with increased incidence of lymph node metastasis [108].

These collective observations predict that the wound–like functions of HMW–HA, LMW–HA, and O–HA are oncogenic in the context of breast cancer but are chronically sustained, which culminates in disease progression rather than resolution. However, additional analyses of which HMW–HA, LMW–HA, and O–HA polymers exert immune and fibrogenic functions [110,129], the cell types that are targeted by these polymers and their functional consequence to cancer cells is needed.

## 4. Roles of HA Receptors in De–Coding HA Polymer Size

### 4.1. Cutaneous Wound Repair

To date, a mechanistic understanding of how immune and mesenchymal cells detect and respond to differences in HA polymer size during physiological and disease processes is not well understood [59]. CD44 is constitutively expressed in skin cells and performs multiple functions during tissue injury, which can either promote or resolve inflammation. This multifunctional property is likely context dependent, since CD44 binds to multiple sizes of HA polymers. Total or basal keratinocyte–targeted (K14) loss of CD44 does not detectably affect uninjured skin architecture [130,131] but embryonic deletion of CD44 mildly increases the inflammatory phase of excisional repair. Thus, neutrophils, M1 and M2 macrophages, and CD3+ T cells are slightly but significantly enhanced, and this increase is accompanied by elevated IL1B and IL4 expression. In contrast to its mild immunogenic effects, genomic loss of CD44 substantially alters the temporal profile and wound distribution of SMA^+^ and FAP^+^ fibroblasts subsets resulting in increased fibro–proliferation and scar formation relative to wildtype wounds. These results predict that CD44 signaling suppresses fibroplasia and may contribute to the anti–fibrotic impact of HMW–HA.

In contrast to CD44, RHAMM is not constitutively expressed in skin but is upregulated with excisional injury [132,133] and preferentially binds to LMW and O–HA [98,134,135]. Genomic *Rhamm*–loss and RHAMM function–blocking reagents robustly reduce inflammation and fibrosis [29,75,81,98,133,136]. Specifically, *Rhamm*–loss, RHAMM mimetic peptides, which bind to and sequester LMW– and O–HA to limit access of these polymers to RHAMM [75], and RHAMM blocking antibodies alter fibroblast heterogeneity, reduce wound macrophage number/cytokine expression, blunt fibroplasia, and promote expression of dermal markers such as Tenascin–C for regenerative repair [137,138]. The immunogenic and fibrogenic effects of RHAMM match closely with those of HA fragments. For example, RHAMM expression is required for dermal fibroblast migration and wound macrophage influx–promoting effects of 6mer O–HA [98].

### 4.2. Breast Cancer Progression

Several studies show that the functions of HA during tumorigenesis are associated with the expression and display of HA receptors on tumor and host cells. For example, the invasive/metastatic triple negative breast cancer cell subsets that bind to high levels of HA display high levels of CD44 and RHAMM [128]. CD44 is widely used as a marker for breast tumor–initiating cells [139,140] and experimental analyses show that CD44 contributes to the pro–tumorigenic behaviour of breast cancer cells by stimulating cell proliferation, migration, invasion, and plasticity [141,142]. These effects of CD44 expression are linked to activation of pro–tumorigenic signaling pathways via partnership with growth factor receptors and RHAMM. The oncogenic functions of CD44 are complex and are affected by posttranslational modification and alternative splicing of this transmembrane protein as well as its epigenetic regulation of gene expression. For example, CD44 mediates uptake of iron–bound hyaluronan that supports the iron–dependent demethylation of histones and upregulation of cell plasticity genes [143]. Intriguingly, CD44 can suppress or support tumorigenicity in a context–dependent manner. Xenograft studies of human cell lines show a role for CD44:HA interactions in promoting breast cancer progression [120] while conversely, lung metastasis is enhanced rather than suppressed in a CD44^–/–^ mouse model of mammary gland susceptibility [144]. These experimental differences suggest that CD44 can be oncogenic or tumor suppressive depending upon the host immune microenvironment. Despite these known oncogenic and tumor–suppressing functions of CD44, this HA receptor is being explored as a therapeutic target, imaging agent and tumor marker in breast and other cancers [145,146]. Successful use of its clinical potential, particularly for therapeutic targeting, will likely require a greater mechanistic understanding for the biological and molecular contexts of the tumor–supporting vs. tumor–suppressing properties of CD44.

High RHAMM expression in tumor cell subsets is a marker for increased peripheral metastasis and poor outcome [147]. The wound–like functions of RHAMM that contribute to breast cancer malignancy include increased cell migration, invasion, and proliferation [8]. Other functions which may or may not be linked to its HA binding properties include effects on cellular polarity, plasticity, genomic stability, chemo–resistance, and de–regulation of oncogenic driver pathways. The tumorigenic consequence of de–regulated RHAMM expression is influenced by the molecular subtype. For example, whereas RHAMM expression is increased in most breast cancers compared to adjacent normal breast tissue, luminal A subtype breast cancers displays a relatively low RHAMM expression compared to other breast cancer types [148] and *RHAMM* knockdown in cell lines derived from this subtype increases rather than decreases migration and metastasis. However, blocking RHAMM signaling in triple negative breast cancer blunts invasion and metastasis and ablation of the HA binding capability of RHAMM destroys its transforming potential [149]. These results and the restricted expression of RHAMM in normal tissues, which contrasts with constitutive and widespread CD44 expression, predict that RHAMM is an attractive potential cancer therapy target for the breast cancer subtypes that use this HA receptor to promote invasion and metastasis.

## 5. Conclusions

In summary, the dynamic changes of HA concentration and fragment size distribution in the remodelling microenvironments of wounds and breast tumors provide cells with important contextual information, that promotes but also limits specific immune and fibroblast functions. This contextual information is “interpreted” by a dynamic expression of HA receptors in particular CD44 and RHAMM, which couple signaling pathways that control cellular migration, invasion, proliferation, and immune regulation required for both efficient wound repair and metastatic spread of tumors. Despite this functional complexity, the medical and cosmetic use of the HA polymer is a growing industry, and in particular experimental studies predict that targeting HA synthesis, hyaluronidases, and HA receptors has enormous therapeutic potential for improving wound repair and management of cancer. For example, application of HMW–HA and its modified derivatives improve cutaneous wound repair [25,150]. HMW–HA is also being developed in experimental models and clinical trials to target CD44 for both imaging and delivery of therapeutics to cancer stem cells [145,151]. Conversely, blocking HA synthesis with 4MU reduces tumor spread [152,153,154,155], increases exposure of tumor cell HER2 for PET imaging of tumors [156] and sensitizes tumor cells to trastuzumab [157]. Modifying the remodeling tumor microenvironment using stabilized hyaluronidases to remove HMW–, LMW–, and O–HA is a novel method for improving delivery of therapeutic drugs to multiple cancers including breast [158,159,160,161]. Targeting HA receptors has also met with success in moderating fibrotic wound repair and managing cancer. For example, RHAMM peptide mimetics that bind to LMW–HA and O–HA reduce fibroplasia in bleomycin–induced lung and skin injury, and promote a regenerative repair in excisional wounds [29,75]. Finally, CD44–HA interactions are actively investigated for their therapeutic potential in particular as a target for HA–based drug formulations [162]. As well, CD44 monoclonal antibodies are being assessed in pre–clinical and clinical trials for both imaging and treating cancers and cancer stem cells that overexpress CD44 [145,146,163,164]. As knowledge of the hyaluronome’s multifunctionality deepens, the number of medical uses, particularly in the realm of wound repair and cancer, will undoubtedly increase.
